# The Accuracy of CBCT in the Detection of Dens Invaginatus in a Tunisian Population

**DOI:** 10.1155/2021/8826204

**Published:** 2021-01-25

**Authors:** Rym Mabrouk, Latifa Berrezouga, Nadia Frih

**Affiliations:** ^1^ABCDF Laboratory, Endodontics and Restorative Dentistry, Hospital of Charles Nicolle, Tunis, Tunisia; ^2^Immunology Restorative Dentistry, Endodontics Faculty of Dental Medicine, Monastir, Tunisia; ^3^Forensic Dentistry and Head of Service of Dental Medicine Department, Hospital of Charles Nicolle, Tunis, Tunisia

## Abstract

**Objective:**

To assess the prevalence and characteristics and to classify the type of dens invaginatus (DI), in anterior teeth, basing on 200 Tunisian patients' cone-beam computed tomography. *Material and Methods*. A retrospective study was performed using CBCT images of 200 Tunisian patients. Maxillary and mandibular anterior teeth were evaluated for the presence and characteristics of dens invaginatus. Results were reported as frequencies, means ± SD. Statistical evaluation of the presence of DI related to gender was performed by the Pearson chi-square test.

**Results:**

Dens invaginatus was observed in 42 out of 4945 examined teeth, with a prevalence of 0.85%.The anomaly affected especially maxillary lateral incisors (*p* ≤ 0.001). DI location was unilateral in 36% and bilateral in 64%. Type II DI was the most commonly observed (47.61%), followed by type I (30.95%) and type III (21.42%). Apical periodontitis (AP) was mostly seen in type III DI, with a periapical index (PAI) varying from 3″ to 5 E″.The maxillary lateral incisors seem to be the most affected anterior teeth. The symmetric occurrence of DI was observed with a statistically significant difference (*p* ≤ 0.001).

**Conclusion:**

Within the limits related to the present study, DI detected by CBCT is a rare anomaly that could be associated with AP. Maxillary anterior teeth should be bilaterally examined for DI in the absence of clinical symptoms.

## 1. Introduction

Dens invaginatus (DI) is a development anomaly that occurs from the infolding of the enamel organ into the dental papilla before the calcification of dental tissue [1, 2]. This dental anomaly was described for the first time by Ploquet in 1794, who discovered DI in a whale's teeth, then reported by a dentist named Socrates in 1856 in human teeth [1]. External forces on the tooth germ during development such as adjacent tooth germs, trauma, and infection, focal growth retardation on the tooth bud, focal growth acceleration on the tooth bud, and restriction of the dental arch on the enamel organ are the probable causal elements for the etiology of invaginated teeth [3, 4]. Many terms were attributed to this anomaly: dens in dente (Bush 1897), dens invaginatus, dilated composite odontome (Hunter 1951), ectasiant or gestant odontoma (Colby 1956), dentoid in dente, deep foramen caecum, and tooth inclusion [1, 5, 6].

Clinically, abnormal crown morphology and features were observed and described in the literature in deep relation with DI including peg-shaped formation, increased labiolingual and mesiodistal diameter, and conical morphology. The presence of an enlarged palatal cingulum or talon cusp can be also detected on the palatal side of the tooth [3, 7]. The invagination may be limited to the pulp chamber, or it may extend to the root. Different classifications were proposed in order to assess the characteristic of DI and to establish management guidelines of different type of DI [8].

The diagnosis of DI is also based on oral radiography. Two‐dimensional imaging such as periapical radiographs or panoramic radiographs are the most commonly used radiographic methods in endodontics. However, these conventional radiographs are insufficient in most cases of DI as they show only a 2D view of a complex anatomy [9, 10].

Cone-beam computed tomography (CBCT) is an extraoral imaging system which produces 3-dimensional scans of the maxillofacial skeleton, including the teeth and their surrounding tissue. CBCT has become a valuable tool for diagnosing and managing of endodontic problems, as well as for assessing root fractures, apical periodontitis, resorptions, perforations, root canal anatomy, and tooth invagination, overcoming the limitations of conventional radiography [11, 12].

The use of CBCT images is crucial to assess the DI type and to observe the extent within the root canal system with high precision. CBCT is a valuable aid in providing additional information for the diagnosis and management of such complex endodontic malformation compared to using intraoral radiographs alone [13].

Dens invaginatus is generally underdiagnosed and in most of cases is detected by chance on radiograph. In addition, there are limited studies reporting the prevalence of DI among population. Therefore, the purpose of the present retrospective study was to investigate the prevalence and distribution of this anomaly in a Tunisian population and to explore its different features and types using cone-beam computed tomography.

## 2. Materials and Methods

### 2.1. Type of the Study

This retrospective descriptive study was conducted based on 200 cone-beam computed tomography images collected from a private department of radiology located in Tunis. CBCT scans were obtained anonymously of patients of both sexes who were referred due to various indications for CBCT scans: impacted teeth, dental implants, orthodontics, maxillofacial surgery, oral pathology, orthognathic surgery, and endodontic treatment within a period between December 2017 and August 2019. All scans used in the present study were taken for clinical reasons, and no patient underwent a CBCT scan exclusively for this study.

### 2.2. CBCT Machine Characteristics

All of the CBCT scans were obtained with NewTom CBCT machine (NewTom Cone Beam 3D imaging units, QR, Verona, Italy), NNT viewer version 7.2 (installation package 7.2.0). The acquisition process was performed by an experienced radiologist according to the manufacturer's recommended protocol. Coronal and sagittal cross-sectional images were transmitted to a personal computer in the digital imaging and communications in medicine (DICOM) format and reconstructed into multiplanar images using the DICOM viewer: NNT viewer (QR Srl–Via Silvestrini, Verona, Italy).

### 2.3. Inclusion Criteria

Only high quality scans were included in this study. CBCT scans with the presence at least of one central and one lateral upper incisor were retained for analysis. Noninclusion criteria were poor quality CBCT images, number of teeth <18, incomplete records, artifacts caused by metallic implants or prosthetic restoration, and scans with low resolution and patient movement during imaging. Maxillary and mandibular anterior teeth were evaluated based on CBCT scans to determine the type of dens invaginatus using Oehlers' classification [7]. Type l: the invagination is enamel lined confined within the crown of the tooth and does not extend beyond about the level of the external amelocemental junction. Type II: the enamel-lined invagination invades into the root but remains confined within it as a blind sac. Type III: the invagination penetrates deeply through the root and “bursts” apically or laterally at a foramen, sometimes referred to as a “second foramen,” in the root.

### 2.4. CBCT Examination

Cone-beam computed tomography scans were examined, separately by two experienced endodontists, for the presence of DI to reduce the interexaminer errors. Agreements were 100% between the two examinations for the presence of DI, indicating the diagnostic reproducibility.

### 2.5. Statistical Analysis

Results were reported as frequencies, means ± SD. The Pearson chi-square test was used to establish potential relation between DI and different studied variables: DI and gender, DI and invaginated lateral incisors, invagination and bilateral affection, and invagination and periapical lesions. Statistical analysis was performed using IBM SPSS Statistics 21.0 for Windows. The level of significance for all tests was set at *P* < 0.05.

## 3. Results

The study population was composed of 200 patients, 98 males (49%), and 102 females (51%) who underwent CBCT imaging for different reasons. The average of age was 44, 92 ± SD 15.363 ranged from 16 to 89 years old. CBCT scans showed that 25 out of 200 patients have at least one DI with a percentage of 12.5%. Males seemed to have more invaginated teeth (15 males out of 25 patients, 60%) than females (10 out of 25; 40%) with no statistically significant gender difference (*p*=0.24) (Table 1).

Forty-two out of 4945 examined teeth were invaginated teeth (0.85%). DI was observed in 5.46% out of 786 anterior assessed teeth. All invaginated teeth were maxillary anterior teeth, and no DI was observed in mandibular teeth. Assessment of different types of invaginated teeth was based on Oehlers ‘classification on 1957 [7]. The most observed type was type II 47.61% followed by type I 30.95% and finally type III with a percentage of 21.42%. The distribution of DI types is shown in Table 2.

**Figure 1:**
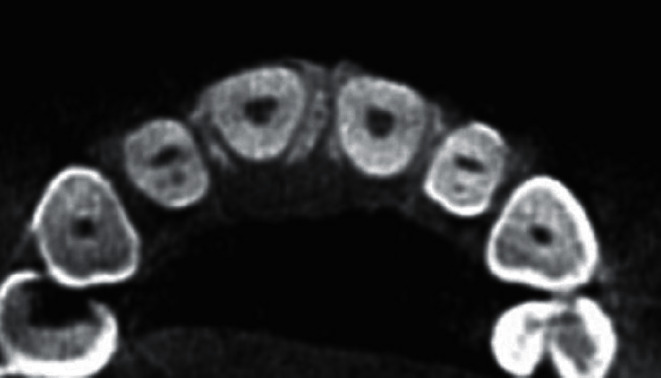
Example of bilateral invagination in lateral maxillary teeth (axial plane).

Nine out of 25 patients with DI had one tooth affected with DI, while 15 patients had bilateral DI (Figure 1) and only one patient had three DI.

**Figure 2:**
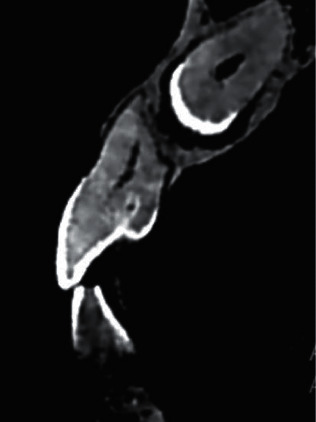
Example of coronal invagination of the left maxillary lateral incisor associated with an impacted maxillary canine (cross-sectional image).

Maxillary central incisors (4 out of 386; 1.03%) and lateral maxillary incisors (38 out of 382; 9.9%) were found to be affected by DI, while none of the posterior teeth in the maxilla neither in the mandible were affected. One case of an impacted maxillary canine associated with a type I of DI was detected (Figure 2).

Lateral maxillary incisors seem to be the most affected teeth with DI (*p* ≤ 0.001) (Table 3).

**Figure 3:**
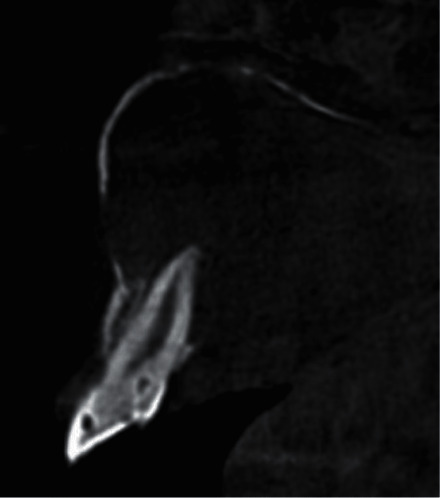
The right maxillary lateral incisor is a type II DI associated with an apical periodontitis PAI index 5 E″ cross-sectional image.

CBCT scans showed two cases of periapical lesions associated with invaginated anterior maxillary teeth type II DI and four periapical lesions related to DI type III. Index score varied from 3 to 5 E & 5 D (Figure 3).

## 4. Discussion

Dens invaginatus prevalence ranged from 0.3% to 10% of teeth and from 0.25 to 26.1% of patients according to several studies [2, 8, 14]. In the present study, the DI was found in 12.5% of individuals examined with no gender difference. Kirzioğlu and Ceyhan [14] reported a low prevalence of 5.90% with no sex-related difference. However, Günduz et al. [15] described a statistically higher prevalence of DI in female patients. The wide variation in reported prevalence may be explained by the different study sample, geographical differences, identification criteria used, diagnostic difficulties, and radiographic methods [14].

Using Oehlers' classification, the prevalence of each type of invagination was reported by Ridell et al. [4] with type I being the most common observed type (79%) while type II (15%) and III (5%) less frequently observed. Cakici et al. [16], also, reported that type I was the most common type of dens invaginatus with a prevalence of 81.25%. However, in the present study, type II (47.61%) was the most observed DI type followed by type I and only 21.42% were type III DI.

The maxillary lateral incisors seemed to be the most affected anterior teeth (90.47%) followed by central maxillary incisors (9. 05%). The appearance of bilateral affection is a common finding by some authors. Kirzioğlu and Ceyhan [14] and Gündüz et al. [15] reported that 82% and 67.5% of the cases were bilateral. In the present study, CBCT scans showed that 15 out of the 25 patients (60%) with DI had bilateral occurrence, while the remaining patients (36%) had unilateral DI and one patient had a bilateral occurrence with three DI in anterior maxillary teeth. The symmetric occurrence of DI was observed in CBCT scans with a statistically significant difference (*p* ≤ 0.001). Dens invaginatus could be associated with other dental anomalies such as gemination, microdontia, macrodontia, impacted tooth taurodontism, and supernumerary tooth. In this study, one case of DI occurred with an impacted maxillary canine. Dens invaginatus affected especially maxillary teeth. No dens invaginatus was detected in the mandibular teeth. These results were consistent with those reported by Kirzioğlu and Ceyhan [14]. However, this finding is not supported by several case reports, which have observed dens invaginatus in mandibular teeth [17]. In the present study, there were no periapical lesions in anterior teeth with types I; however, CBCT scans showed two cases of periapical lesions associated with invaginated anterior teeth type II DI and four periapical lesions related to DI type III. Teeth affected by dens invaginatus were considered to have a poor prognosis, and extraction was advocated as the treatment of choice [2, 8]. Oehlers' system is based on bidimensional radiographic images which do not reveal the real extent and complexity of DI. The use of 2D radiographs to aid in the diagnosis of dens invaginatus is influenced by some factors such as the quality of the radiograph. Furthermore, the angulation of the fllm is particularly important because the presence of an invagination may not be apparent on standard parallel views. In addition, normal conventional radiographs did not provide detailed information concerning the complexity of DI anomaly because of geometric compression of a 3D volume into 2D images that leads to a lack of information. The limitations associated with the use of conventional radiography in the classiflcation and management of dens invaginatus are overcomed with the increasing availability of computerized 3D imaging such as CBCT [18]. CBCT is a valuable imaging technique in determining the access point [19], the path of the invagination, and the possible communication between the invaginated tissue within the pulp or the periodontal tissues. The knowledge of the communication between the pulp and the lumen is relevant from the treatment point of view as it determines whether or not to endodontically treat the pulp chamber. For this reason, in teeth with no other obvious cause for pulp disease, the presence of an invagination should be considered, particularly if other clinical and radiographic features associated with the anomaly are suspected [2, 4, 18]. In case when endodontic treatment is intended and particularly in teeth with severe invaginations, CBCT allows a 3D visualization of the true morphology of the invagination, the type of the abnormality, and the real extent and allows the clinician better treatment management [19].

## 5. Conclusion

The present paper is the first study investigating the prevalence and distribution of DI using CBCT in Tunisia. Maxillary anterior teeth must be carefully bilaterally examined for the presence of DI even in the absence of clinical symptoms. If DI is suspected, clinical examination is essential; careful radiographic interpretation is also required. To confirm the suspicion of DI, an additional radiographic examination should be performed; CBCT is adequate for this purpose.

## Figures and Tables

**Table 1:** Distribution of subjects with dens invaginatus.

	Female	Male	*p* value
Subjects with DI	10 (40%)	15 (60%)	0.240
Subjects without DI	92	83	
Total	102	98	

DI: dens invaginatus; *p*: results of Pearson's chi-square test comparing the gender distribution.

**Table 2:** Distribution of the type of dens invaginatus (DI) in anterior maxillary teeth.

		Number of teeth with DI	Prevalence (%)
Type of DI	Type I	13	30.95
	Type II	20	47.61
	Type III A	4	9.52
	Type III B	5	11.90
	Total	42	100

**Table 3:** Distribution of dens invaginatus according to location.

Teeth	Number of affected teeth = 42	
Maxillary	Right	Left
Lateral incisor	16 (38.095%)	22 (52.38%); *p* ≤ 0.001
Total 38 (90.47%); *p* ≤ 0.001
Central incisor	2 (4.76%)	2 (4.76%)
Total 4 (9.52%)

## Data Availability

The data that support the findings of this study are available on request from the corresponding author.
